# Using Chinese Body Constitution Concepts and Measurable Variables for Assessing Risk of Coronary Artery Disease

**DOI:** 10.1155/2019/8218013

**Published:** 2019-09-16

**Authors:** Yi-Chia Huang, Chien-Jung Lin, Shu-Meng Cheng, Chi-Kuei Lin, Sunny Jui-Shan Lin, Yi-Chang Su

**Affiliations:** ^1^Graduate Institute of Chinese Medicine, College of Chinese Medicine, China Medical University, Taichung 40402, Taiwan; ^2^Department of Chinese Medicine, Tri-Service General Hospital, National Defense Medical Center, Taipei 11490, Taiwan; ^3^Chinese Medical Advancement Foundation, Taipei 10041, Taiwan; ^4^Department of Internal Medicine, Tri-Service General Hospital, National Defense Medical Center, Taipei 11490, Taiwan; ^5^Chander Clinic, Taipei 10646, Taiwan; ^6^School of Chinese Medicine, College of Chinese Medicine, China Medical University, Taichung 40402, Taiwan; ^7^National Research Institute of Chinese Medicine, Ministry of Health and Welfare, Taipei 11221, Taiwan

## Abstract

**Background:**

Identifying patients with high risk of coronary artery disease (CAD) is often difficult in outpatient clinic settings. This study aimed to explore if the measurement of body constitution can be adopted to predict the risk of CAD diagnosis. The objective of this study is to conduct a prospective observational study and a case-control study to answer the research question.

**Study Design:**

Part 1 (prospective observational study): a total of 143 patients with chest pain and admitted to receive cardiac catheterization were enrolled, and 108 of them were diagnosed with CAD. Part 2 (case-control study): the above 108 CAD patients and 476 healthy controls matched by age and gender from the participants of Taiwan Biobank were adopted for comparison.

**Main Outcome Measures:**

The body constitution of both patients and healthy controls were measured by the Body Constitution Questionnaire (BCQ). Each one received scores of *Yang-Xu* (*Yang-deficiency*), *Yin-Xu* (*Yin-deficiency*), and *Stasis*. These 3 scores together with demographic characteristics and CAD risk factors were used in the logistic multiple regression model to predict the risk of CAD.

**Results:**

(Part 1) No difference was found between the scores of *Yang-Xu*, *Yin-Xu*, and *Stasis* between the patients with and without CAD. (Part 2) The scores of *Yang-Xu*, *Yin-Xu*, and *Stasis* of the CAD patients were significant higher those of the healthy controls. *Yang-Xu* and *Stasis* scores were obtained with age, BMI, and hypertension in the model with prediction rate 89.0%. The area under receiver operating characteristic curve of this model was 0.896.

**Conclusions:**

This study is the first to apply Chinese body constitution concepts and measurable variables to assess the risk of having CAD of the patients with chest pain prior to receiving cardiac catheterization. The higher scores of *Yang-Xu* and *Stasis* were found to be risk factors. Our results revealed that BCQ has the potential to be a first-line diagnostic tool for patients with chest pain to facilitate early recognition and diagnosis of CAD.

## 1. Introduction

Coronary artery disease (CAD) remains a significant global public health burden. Identifying patients with high-risk CAD before diagnostic testing can help ensure these patients receive closer follow-up or early cardiac catheterization [1]. However, early recognition and diagnosis remain challenging. Many physicians experience the clinical pressure to accurately rule out CAD as a cause of chest pain.

Throughout the growth of western medicine from Hippocrates, body constitution has been a leitmotiv of human diseases. Research of body constitution offers an approach to the study of the “host factor” of the disease development. Many associations are found among somatotypes and human illness, particular in CHD where the highest risks are among endomorphic mesomorphs. However, these findings are limited to western groups [2]. In traditional Chinese medicine (TCM), body constitution is also the core of medical theories [3]. It reflects the uniqueness of individuals' physiological features, their susceptibility to pathogenic factors, and tendency to certain type of pathological changes [4].

In TCM, human body is considered as healthy when the dynamic of *Yin* and *Yang* is balanced. The definition of *Yin* is the materials that perform physiological function, e.g., tissues, blood, and body fluid, while *Yang* is the energy which maintains physiological function, e.g., energy for heart contraction, breathing, and digestion. In balanced status, *Yang* pushes *Yin* smoothly throughout the whole body without any stagnation. Under the influence of behaviour and aetiology factors, ones' energy and/or materials will decrease. Then, the individual will develop *Yang-Xu* (Yang-deficiency) and/or *Yin-Xu* (Yin-deficiency) constitution. Besides, the behaviour and aetiology factors may let the flow of energy becomes stagnant which will result in production of watery phlegm or static blood [4–6]; then, the individual will develop *Stasis* constitution. The unbalanced constitutions can represent an individual's susceptibility to and recurrences of specific diseases, especially chronic diseases and their complications. For example, type 2 patients with stasis constitution had a higher prevalence rate of the complication peripheral arterial disease [7], and diabetic retinopathy was more prevalent in patients with *Yang-Xu* constitution [8].

The main manifestation of CAD “chest pain” resembles the description of “chest obstruction (胸痹)” recorded in a Chinese medical classic *Jingui Yaolue* (金匱要略) [9]. In modern medicine, CAD is considered to be caused by stenosis or obstruction in coronary arteries, while in TCM, chest pain is the result of *Yang-deficiency*. The decreased energy level results in failure to maintain normal blood circulation and leads to the formation of phlegm and static blood, which resembles the pathogenesis of atherosclerosis. The correlation between CAD and the constitution of TCM was reported by Xu et al. [10], and their findings can verify the abovementioned pathogenesis of CAD in TCM.

TCM physicians generally determine a person's body constitution by analysing the information gathered by four examinations (i.e., inspection, listening and smelling, inquiry, and palpation). However, these methods are subjective and lack consistency among different physicians. The body constitution questionnaire (BCQ) [4–6, 11, 12] was developed to provide an objective tool that meets the scientific research requirements. BCQ is a valid and useful tool for evaluating the *Yang-Xu*, *Yin-Xu*, and *Stasis* constitution. This instrument is also a tool for integrating western and Chinese medicine and has been adopted in the clinical studies on patients of diabetes [7, 8, 13, 14], breast cancer [15, 16], schizophrenia [17], and women with menopausal symptoms [18], perinatal women [19, 20], and healthy group [21].

Optimal management of patients with chest pain relies on the prognostic information provided by diagnostic testing. Invasive coronary angiography (ICA), also called cardiac catheterization, is considered the gold standard for diagnosing CAD. However, given increased risks and cost, noninvasive cardiac testing is a more appropriate first-line diagnostic step. The aim of this study is to explore if the score of *Yang-Xu*, *Yin-Xu*, and *Stasis* constitution measured by BCQ can help to predict the CAD risk and inform case decision.

## 2. Materials and Methods

### 2.1. Study Design and Population

The study design is composed of two parts.

#### 2.1.1. Part 1: Prospective Observational Study

The study participants were stable symptomatic outpatients without known CAD, who were admitted to the cardiology ward of Tri-Service General Hospital, Northern Taiwan, to receive cardiac catheterization for further evaluation. The inclusion criteria were as follows: (1) age > 18 years; (2) prior hospital visits because of chest pain or angina pectoris; (3) admission for cardiac catheterization deemed necessary by cardiologists. The exclusion criteria were as follows: (1) having the history of acute myocardial infarction, arrhythmia, valvular heart disease, cancer, severe infection, or severe mental illness and pregnancy; (2) inability to complete the questionnaire.

The study participants were diagnosed having CAD by cardiac catheterization. Then, the scores of *Yang-Xu*, *Yin-Xu*, and *Stasis* constitution were compared between the patients with and without CAD.

*(1) Sample Size Calculation*. The study was set to evaluate the correlation between the CAD diagnosis (a dichotomous outcome) and the scores of *Yang-Xu*, *Yin-Xu*, and *Stasis*. The sample size was estimated by a commonly accepted rule: *N*=10*∗k*/*p*=10*∗*3/0.25=120, where *k*=3, the number of variables, and *p*=0.25, the smallest of the proportions of negative or positive events (non-CAD cases in this study) in the study (CAD) population. The rule is based on the work of Peduzzi et al. [22].

#### 2.1.2. Part 2: Case-Control Study

This was performed to compare the scores of *Yang-Xu*, *Yin-Xu*, and *Stasis* constitution between CAD patients and the healthy controls using the data from the Taiwan Biobank (TWB) [23]. TWB aims to build a nationwide research database that integrates genomic/epigenomic profiles, lifestyle patterns, dietary habits, environmental exposure history, and long-term health outcomes of 300,000 residents of Taiwan. The data of the participant who were enrolled by TWB from different cities in Taiwan between 2008 and 2015 and had completed BCQ were adopted as the study group in this case-control study.

*(1) Sample Size Calculation*. Based on the number of CAD patients in the observational study, the healthy cases were matched by gender and age (controls matched age restricted within ± 2 y/o) with a ratio of 5 : 1 from the adopted study group.

### 2.2. Measurements

#### 2.2.1. Diagnosis of CAD

CAD diagnosis was assessed by cardiologists using angiographic images obtained during cardiac catheterization. The diagnosis of CAD was confirmed by the presence of at least one artery with at least 50% stenosis [24].

#### 2.2.2. Scores of *Yang-Xu*, *Yin-Xu*, and *Stasis* Body Constitution

Participants' body constitution was measured by the BCQ, which is a reliable, valid questionnaire and developed according to the Chinese body constitution theories. BCQ consists of 3 independent constitution subscales, including (1) 19 items of *Yang-Xu* (*Yang-deficiency*) [4, 11], (2) 19 items of *Yin-Xu* (*Yin-deficiency*) [5, 12], and (3) 16 items of *Stasis* (*phlegm stasis*) [6]. Because some items overlapped in the 3 subscales, BCQ is composed of 44 items and using a 5-point Likert scale from 1 (never happened) to 5 (always happens). The final score of each constitution is calculated by summing the scores of all items of each subscale, with a higher score implying a greater deviation from the balanced constitution. That is, the higher the score of *Yang-Xu*, the more deficient the *Yang* (energy) is.

Cronbach's *α* and intraclass correlation of BCQ 0.62–0.88 and >0.7, respectively, together with high content validity (0.73–1.00), construct validity, and test-retest reliability [4–6, 11, 12]. Besides, BCQ also demonstrates high discriminant validity (i.e., a *Z* score of 3.3636 to 10.026) and reliability (i.e., Cronbach's *α* of 0.85–0.92) [25].

### 2.3. Study Procedures

The patients were admitted in the afternoon one day before cardiac catheterization. In addition to the systolic and diastolic blood pressure (SBP and DBP) measurement, blood sampling for fasting plasma glucose (FPG), total cholesterol, triglyceride (TG), and low-density cholesterol (LDL) measurement, if required by the patients' cardiologists for clinical diagnoses, were also performed and recorded. The medical history of the patients was collected, e.g., age, sex, height, weight, and related comorbidity (including diabetes, hypertension, and hyperlipidemia). Then, researchers explained the study purpose and procedure to the patients, who then signed the consent form and completed the BCQ. Researchers checked every completed questionnaire to make sure that all the questions were answered. Subsequently, the questionnaire data were entered into a software program to calculate the scores of *Yang-Xu*, *Yin-Xu*, and *Stasis*.

On the next day, the patients received cardiac catheterization. Angiographic images of the coronary arteries were acquired and adopted to determine whether the patients had CAD by cardiologists who were blind to the results of BCQ measurements. The patients were then divided into CAD and non-CAD group according to the diagnosis confirmed by cardiologists.

### 2.4. Ethical Considerations

Prior to implementing the observational study, the study protocol and the participant consent form were submitted to the Institutional Review Board of Tri-Service General Hospital and being approved (number: TSGHIRB 100-05-016). As for the case-control study, the protocol was also approved by the TWB (numbers: TWBR10509-04 and TWBR10603-06).

### 2.5. Data Analysis

Patient demographics, cardiovascular risk factors, and scores of *Yang-Xu*, *Yin-Xu*, and *Stasis* constitution were examined as potential predictors of high-risk CAD. Descriptive statistics for continuous variables are presented as means and SDs, and categorical variables are presented as frequencies and percentages. Comparisons of characteristics between patients of CAD and non-CAD group and between CAD patients and the healthy controls performed by Student's *t*-test for continuous variables and a *χ*^2^ test or Fisher's exact test in cases of low cell counts for categorical variables.

Demographic characteristics, risk factors, and scores of *Yang-Xu*, *Yin-Xu*, and *Stasis* constitution were used in a logistic multiple regression model to predict the risk of CAD (*Part 1: observational study*). A multivariate logistic regression analysis was performed using backward selection to explore the risk factors among the scores of *Yang-Xu*, *Yin-Xu*, and *Stasis* along with other CAD risk factors (e.g., age, gender, body mass index (BMI), history of hypertension, diabetes, and hyperlipidemia) (*Part 2: case-control study*). Multivariate logistic regression was performed with two models: model 1 in which only CAD risk factors, e.g., age, gender, body mass index (BMI), history of hypertension, diabetes and hyperlipidemia were analyzed using the enter method and model 2 in which the candidate predictors included the above CAD risk factors along with the scores of *Yang-Xu*, *Yin-Xu*, and *Stasis* constitution and were analyzed using backward selection. The final models are presented with an odds ratio, 95% CI, and *P* value for each predictor. Subsequently, areas under the receiver operating characteristic curves (ROCs) of model 1 and model 2 were compared to see if the body constitution variables can increase the prediction rate of CAD diagnosis.

Statistical calculations were performed using software package SPSS 20.0 (SPSS Inc., Chicago, IL). All comparisons were two-tailed; *P* < 0.05 was regarded as statistically significant.

## 3. Results

### 3.1. Prospective Observational Study

#### 3.1.1. Comparison of Demographic Characteristics between Patients with and without CAD

A total of 143 patients were enrolled. Among them, 108 (75.52%) received a CAD diagnosis after cardiac catheterization and was assigned as the CAD group; while the other 35 patients without CAD diagnosis were assigned as non-CAD group. A higher proportion of male was found in both CAD and non-CAD groups. The average age of the CAD group was significantly higher than that of the non-CAD group (59.96 ± 9.44 versus 49.56 ± 15.29 years, *P* < 0.01). No significant differences were observed in male proportion, body mass index (BMI), systolic and diastolic blood pressure, and the scores of *Yang-Xu*, *Yin-Xu*, and *Stasis* constitution.

Regarding related comorbidity, the prevalence of diabetes was significantly higher in the CAD group than in the non-CAD group (34.3% versus 5.7%, *P* < 0.01). Among the biochemistry profiles assessed, though the values of FPG, total cholesterol, TG, and LDL of the CAD group were higher than non-CAD group, only FPG reached significant differences (Table 1).

#### 3.1.2. Higher Score of *Yang-Xu* as Risk Factor for CAD

In the final model, *Yang-Xu* score and *Yin-Xu* score were obtained together with age and diabetes as prediction factors (Table 2). It was revealed that the increase in the *Yang-Xu* score increased patients' CAD diagnosis probability (OR: 1.11, 95% CI: 1.01–1.23, *P*=0.04). By contrast, an increase in the *Yin-Xu* score decreased patients' CAD diagnosis probability (OR: 0.89, 95% CI: 0.80–0.98, *P*=0.02).

### 3.2. Case-Control Study

#### 3.2.1. Comparison of Demographic Characteristics between CAD Patients and Healthy Controls

The number of healthy cases matched from TWB was 476. The body mass index (IBM), proportion of having comorbidities (hypertension, diabetes, and hyperlipidemia), and the scores of *Yang-Xu*, *Yin-Xu*, and *Stasis* constitution were significantly higher in the patients with CAD diagnosis compared with the healthy controls (Table 3).

#### 3.2.2. Higher Score of *Yang-Xu* and *Stasis* as Prediction Factors for CAD

The prediction rate of model 1 was 82.5%. Among the CAD risk factors, hypertension (OR=4.64, 95% CI: 2.86–7.52, *P* < 0.01) and hyperlipidemia (OR=2.19, 95% CI: 1.33–3.61, *P* ≤ 0.01) had statistical significance (Table 4).

The prediction rate of model 2 was 89.0%. *Yang-Xu* and *Stasis* scores were obtained with the CAD risk factors: age, BMI, and hypertension (Table 5). The score of *Yang-Xu* (OR: 1.08, 95% CI: 1.02–1.15, *P*=0.02) and *Stasis* (OR: 1.16, 95% CI: 1.08–1.26, *P*=0.02) were found to be positively correlated with the prediction of CAD diagnosis, respectively.

**Figure 1:**
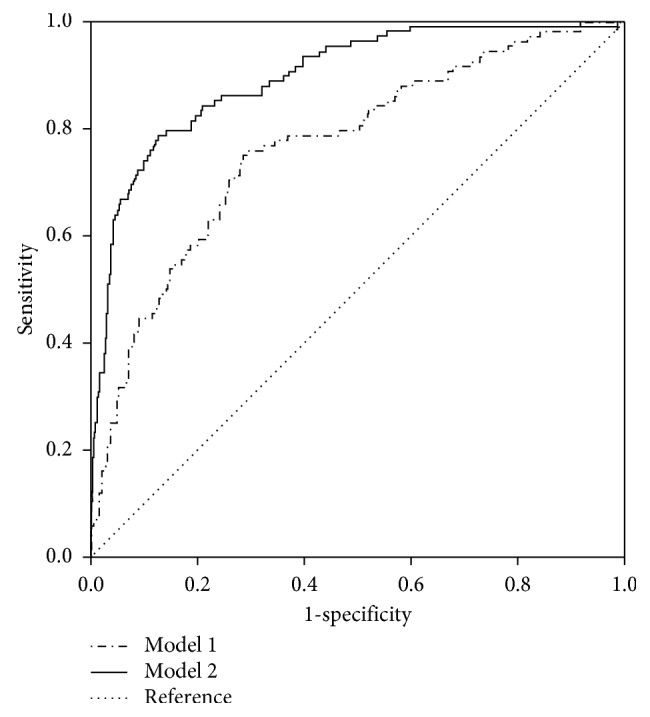
Receiver operator curves comparing model 1 and model 2 for predicting coronary artery disease. Model 1 (CAD risk factors: age, gender, BMI, hypertension, diabetes and hyperlipidemia): AUC = 0.766. Model 2 (the above CAD risk factors and scores of *Yang-Xu* and *Stasis*): AUC = 0.896. AUC: area under the curve.

Finally, the area under ROC (AUC) of model 1 and model 2 was 0.766 and 0.896, respectively. The prediction rate was obviously increased when combining the body constitution scores (Figure 1).

## 4. Discussion

To the best of our knowledge, this study is the first to apply Chinese body constitution concepts and measurable variables to assess the risk of having CAD of the patients with chest pain prior to receiving cardiac catheterization. The higher scores of *Yang-Xu* and *Stasis* were found to be risk factors. Our results revealed that BCQ has the potential to be a first-line diagnostic tool for patients with chest pain to facilitate early recognition and diagnosis of CAD. It takes 10–15 minutes to complete BCQ, which can be done before the patients receive furthermore time-consuming and invasive testing.

Studies have shown that patients with suspected CAD or angina pectoris underwent cardiac catheterization, and no coronary artery stenosis was found in 60% of the patients [26]. Therefore, an easy-applied diagnostic tool is needed to identify the patients with a high risk of having CAD. The results of our study showed that together with the risk factors of CAD, the scores of *Yang-Xu*, *Yin-Xu*, and *Stasis* increased the prediction rate of CAD diagnosis. Thus, the patients can be advised immediately to receive the cardiac catheterization or receive timely treatment to avoid the risk of acute myocardial infarction.

It is considered in TCM that the chest pain is resulted from *Yang-Xu*, in which the lowered driving force is unable to push the *Yin* in blood vessels smoothly; thus, phlegm and static blood formation is formed to cause vessel obstructions [9]. One's body constitution determines the tendency to develop certain types of diseases. Since CAD is a chronic disease that formed slowly over time, we hypothesised that *Yang-Xu* and *Stasis* are the main body constitution as the predisposing factors of CAD and can be adopted as predictors of CAD diagnosis. The results of the case-control study verified this hypothesis.

Higher scores of *Yang-Xu* were found to be the risk factor both in the observation and case-control study, while the correlation of higher score of *Stasis* with the CAD diagnosis was only found in the case-control study. It is understood in TCM theory that once phlegm and *Stasis* are formed, it will continuously affect the Qi-blood circulation. Thus, pain will occur [6]. In Part 1 observational study, there was no correlation found between *Stasis* constitution and the CAD diagnosis, but the score of *Stasis* of the two groups (29.57 ± 9.32 in the CAD group; 30.83 ± 10.02 in the non-CAD group) both exceeded the cutoff point of having the *Stasis* constitution [6]. Therefore, we inferred that patients who suffer from chest pain already have *Stasis* constitution and score of Stasis was not found to be risk factor of CAD in the Part 1 study.

On the contrary, *Yin-Xu* score was found to be negatively associated with CAD in the observational study. The higher the *Yin-Xu* score, the lower the possibility to receive CAD diagnosis. This can be explained by the TCM theories: (1) if *Yin* is enough and accompanied with *Yang-Xu*, the phlegm and static blood will be formed in the vessel [6]; (2) if *Yin-Xu* coexisted with *Yang-Xu*, as long as the *Yang-Xu* status is more obvious than *Yin-Xu* (i.e., the larger the difference between *Yang-Xu* and *Yin-Xu* scores), *Yin* still cannot be driven by *Yang* smoothly. In this condition, CAD will develop easily.

In this study, we also found that among the risk factors of CAD, the odds ratio (OR) of diabetes was very significant in the observational study (Table 2). The influence of diabetes on CAD was reported to be indeed more notable than other risk factors in the other large-scale research on CAD risk factors [27]. Is it possible that diabetes affects one's body constitution status and further influences the formation of CAD? Tsai et al. and Lee et al. adopted BCQ to evaluate the body constitution of patients with type 2 diabetes [8, 13, 14]. The proportion of *Yang-Xu*, *Yin-Xu*, and *Stasis* constitution reported in their studies was 11.7–12.9%, 25.9–27.8%, and 12.8–13.2%, respectively. In our study, among the 42 patients with diabetes, the proportion of *Yang-Xu*, *Yin-Xu*, and *Stasis* constitution was 73.8%, 76.2%, and 69.1%, respectively. We then examined the 40 patients who had both CAD and diabetes, and the proportion of *Yang-Xu*, *Yin-Xu*, and *Stasis* constitution was similar (72.5%, 77.5%, and 67.5%). These proportions were higher than the results of the studies of Tsai et al. and Lee et al. [8, 13, 14]. These differences may be caused by the combination of CAD and diabetes diseases. Since the previous study did not separately evaluate the body constitution of the CAD patients and the number of samples in our study was less than these studies, it may be necessary to conduct a larger-scale study to confirm this inference.

Currently, most CAD-related TCM studies have explored the correlation between patients with different “patterns” and CAD. Their findings are similar to our study; that is, *Yang-deficiency* patterns and Qi*-deficiency* patterns were often identified in patients with CAD, and *Yin-deficiency* patterns were rarely reported [28–30]. While examining the TCM prescriptions for CAD, they can be divided into two main categories: “supplementing Qi” and “dispelling static blood [31].” To sum it up, one's Yang-deficiency results in the formation of static blood is the major pathological mechanism of CAD. According to the results of our study, to improve and to prevent the development of *Yang-Xu* and *Stasis* constitution may prevent the CAD formation, and BCQ can be applied to assess the effects of the preventive intervention. Before applying BCQ in different countries, the translation and cultural adjustment of BCQ need careful consideration [32]. The translated edition must be both conceptually equivalent to the original and easily understood by the people to whom the translated questionnaire is administered.

### 4.1. Study Limitations

This study has the following limitations. First, although BCQ is the optimal, reliable, and valid Chinese body constitution measurement tool, it is relatively new. Thus, the body constitution data of patients with various diseases and the body constitution data of a large-scale population are unavailable. In this study, age, BMI, hypertension, and diabetes were found to be related to CAD diagnosis. However, whether these factors also affect patients' body constitution could not be inferred because of the lack of related body constitution distribution data in Taiwan. Furthermore, it is not acceptable to let healthy people receive cardiac catheterization, which is invasive, under the consideration of research ethics. Therefore, we conducted a case-control study using the data from TWB for comparison.

CAD may cause myocardial ischemia or myocardial necrosis, but not all patients with CAD experience myocardial injuries. Because of the lack of nuclear medicine reports, which can be used to assess patients' cardiomyopathy status, the correlation between body constitution changes and cardiomyopathy could not be analyzed.

The sample size of this study was relatively small and comprised only patients from Tri-Service General Hospital in Northern Taiwan. Thus, the homogeneity of the sample is limited. In addition, disease development is mainly determined by one's body constitution, but the body constitution formation and disease onset are also influenced by factors such as the environment and climate.

## 5. Conclusions

This study is the first study to apply Chinese body constitution concepts and measurable variables to assess the risk of having CAD of the patients with chest pain prior to receiving cardiac catheterization. The higher score of *Yang-Xu* and *Stasis* constitution were found to be risk factors. BCQ is noninvasive, convenient, and inexpensive instrument and may provide prognostic information for health care professionals to identify patients with chest pain who are at a high risk of CAD.

## Figures and Tables

**Table 1:** Comparison of demographic characteristics between patients with and without coronary artery disease.

Variable	CAD (*n*=108)	Non-CAD (*n*=35)	*P* value
Gender (male: *n*, %)	85 (78.7%)	26 (74.3%)	NS
Age (yr)	59.96 ± 9.44	49.56 ± 15.29	<0.01
BMI (kg/m^2^)	26.22 ± 3.95	25.81 ± 4.88	NS
Comorbidity			
Hypertension (*n*, %)	69 (63.9%)	16 (45.7%)	NS
Diabetes (*n*, %)	37 (34.3%)	2 (5.7%)	<0.01
Hyperlipidemia (*n*, %)	45 (41.7%)	11 (31.4%)	NS
SBP (mmHg)	135.28 ± 14.90	131.26 ± 20.52	NS
DBP (mmHg)	80.70 ± 9.58	79.83 ± 10.16	NS
FPG (mg/dl)	147.46 ± 62.35 (97)	116.76 ± 31.45 (29)	<0.01
Total cholesterol (mg/dl)	166.11 ± 34.28 (55)	158.77 ± 24.72 (22)	NS
TG (mg/dl)	136.26 ± 58.07 (57)	131.91 ± 75.39 (22)	NS
LDL (mg/dl)	109.45 ± 31.44 (58)	103.33 ± 24.40 (21)	NS
*Yang-Xu* score	35.38 ± 9.65	35.74 ± 12.08	NS
*Yin-Xu* score	34.06 ± 9.98	35.83 ± 11.28	NS
*Stasis* score	29.57 ± 9.08	30.83 ± 10.02	NS

Continuous data are presented as mean ± SD. Categorical data are presented as number of patients (percentages). *P* < 0.05 being statistically significant. For FPG, total cholesterol, LDL, and TG, the number of people examined is indicated in parentheses. NS: not significant. BMI: body mass index = weight (kg)/height (m^2^). CAD: coronary artery disease; SBP: systolic blood pressure; DBP: diastolic blood pressure; FPG : fasting plasma glucose; LDL: low-density lipoprotein; TG: triglyceride.

**Table 2:** Multivariate analysis of predicting factors for CAD.

Variable	*B*	Wald	Prediction rate: 83.2% odds ratio	95% CI	*P* value
Age	0.07	11.52	1.08	1.03–1.12	<0.01
Gender	−0.93	2.56	0.39	0.13–1.23	NS
BMI	0.05	0.64	1.05	0.93–1.18	NS
Hypertension	−0.08	0.03	0.92	0.36–2.39	NS
Diabetes	2.19	6.61	8.96	1.69–47.67	0.01
Hyperlipidemia	0.30	0.37	1.35	0.51–3.55	NS
*Yang-Xu* score	0.11	4.46	1.11	1.01–1.23	0.04
*Yin-Xu* score	−0.12	5.68	0.89	0.80–0.98	0.02

Multivariate logistic regression analysis was performed by the “backward” method. *P* < 0.05 being statistically significant. NS: not significant. CI: confidence interval.

**Table 3:** Comparison of demographic characteristics between CAD patients and healthy cases.

Variable	CAD (*n*=108)	TWB-HC (*n*=476)	*P* value
Gender (male: *n*, %)	85 (78.7%)	391 (82.1%)	NS
Age (yr)	59.96 ± 9.44	58.05 ± 8.30	NS
BMI (kg/m^2^)	26.22 ± 3.95	24.69 ± 3.16	<0.01
Comorbidity			
Hypertension (*n*, %)	69 (63.9%)	106 (22.3%)	<0.01
Diabetes (*n*, %)	37 (34.3%)	79 (16.6%)	<0.01
Hyperlipidemia (*n*, %)	45 (41.7%)	90 (18.9%)	<0.01
*Yang-Xu* score	35.38 ± 9.65	25.31 ± 5.69	<0.01
*Yin-Xu* score	34.06 ± 9.98	25.57 ± 5.58	<0.01
*Stasis* score	29.57 ± 9.08	20.45 ± 4.55	<0.01

Continuous data are presented as mean ± SD. Categorical data are presented as the number of patients (percentages). *P* < 0.05 being statistically significant. NS: not significant. BMI: body mass index = weight (kg)/height (m^2^). CAD: coronary artery disease; TWB-HC : Taiwan Biobank healthy cases.

**Table 4:** Multivariate analysis of predicting factors for CAD.

Variable	*B*	Wald	Prediction rate: 82.5% odds ratio	95% CI	*P* value
Age	0.02	1.07	1.02	0.97–1.05	NS
Gender	−0.37	1.67	0.68	0.38–1.22	NS
BMI	0.06	2.83	1.06	0.99–1.14	NS
Hypertension	1.53	38.52	4.64	2.86–7.52	<0.01
Diabetes	0.33	1.51	1.39	0.82–2.37	NS
Hyperlipidemia	0.78	9.46	2.19	1.33–3.61	<0.01

Multivariate logistic regression analysis was performed by the“enter” method. *P* < 0.05 being statistically significant. NS: not significant. CI: confidence interval.

**Table 5:** Multivariate analysis of predicting factors (body constitution scores included) for CAD.

Variable	*B*	Wald	Prediction rate: 89.0% odds ratio	95% CI	*P* value
Age	0.05	6.36	1.05	1.01–1.09	0.01
Gender	0.61	2.50	1.84	0.86–3.91	NS
BMI	0.08	4.05	1.09	1.00–1.18	0.04
Hypertension	1.46	23.68	4.29	2.39–7.70	<0.01
Diabetes	0.24	0.51	1.27	0.66–2.42	NS
Hyperlipidemia	0.58	3.38	1.78	0.96–3.29	NS
*Yang-Xu* score	0.08	5.97	1.08	1.02–1.15	0.02
*Stasis* score	0.15	14.22	1.16	1.08–1.26	<0.01

Multivariate logistic regression analysis was performed by “backward” method. *Yang-Xu* score and *Stasis* score: variables measured by Body Constitution Questionnaire (BCQ). *P* < 0.05 being statistically significant. NS: not significant. CI: confidence interval.

## Data Availability

The data used to support the findings of this study are available from the corresponding author upon request.
